# *BACH1 *Ser919Pro variant and breast cancer risk

**DOI:** 10.1186/1471-2407-6-19

**Published:** 2006-01-24

**Authors:** Pia Vahteristo, Kristiina Yliannala, Anitta Tamminen, Hannaleena Eerola, Carl Blomqvist, Heli Nevanlinna

**Affiliations:** 1 Departments of Obstetrics and Gynecology, Helsinki University Central Hospital, Helsinki, Finland; 2 Department of Medical Genetics, University of Helsinki, Helsinki, Finland; 3 Departments of Oncology, Helsinki University Central Hospital, Helsinki, Finland; 4 Department of Oncology, Uppsala University Hospital, Uppsala, Sweden

## Abstract

**Background:**

BACH1 (BRCA1-associated C-terminal helicase 1; also known as BRCA1-interacting protein 1, BRIP1) is a helicase protein that interacts *in vivo *with BRCA1, the protein product of one of the major genes for hereditary predisposition to breast cancer. Previously, two *BACH1 *germ line missense mutations have been identified in early-onset breast cancer patients with and without family history of breast and ovarian cancer.

In this study, we aimed to evaluate whether there are *BACH1 *genetic variants that contribute to breast cancer risk in Finland.

**Methods:**

The *BACH1 *gene was screened for germ line alterations among probands from 43 Finnish *BRCA1/2 *negative breast cancer families. Recently, one of the observed common variants, Ser-allele of the Ser919Pro polymorphism, was suggested to associate with an increased breast cancer risk, and was here evaluated in an independent, large series of 888 unselected breast cancer patients and in 736 healthy controls.

**Results:**

Six *BACH1 *germ line alterations were observed in the mutation analysis, but none of these were found to associate with the cancer phenotype. The Val193Ile variant that was seen in only one family was further screened in an independent series of 346 familial breast cancer cases and 183 healthy controls, but no additional carriers were observed. Individuals with the *BACH1 *Ser919-allele were not found to have an increased breast cancer risk when the Pro/Ser heterozygotes (OR 0.90; 95% CI 0.70–1.16; p = 0.427) or Ser/Ser homozygotes (OR 1.02; 95% CI 0.76–1.35; p = 0.91) were compared to Pro/Pro homozygotes, and there was no association of the variant with any breast tumor characteristics, age at cancer diagnosis, family history of cancer, or survival.

**Conclusion:**

Our results suggest that the *BACH1 *Ser919 is not a breast cancer predisposition allele in the Finnish study population. Together with previous studies, our results also indicate that although some rare germ line variants in *BACH1 *may contribute to breast cancer development, the contribution of *BACH1 *germline alterations to familial breast cancer seems marginal.

## Background

BACH1 (BRCA1-associated C-terminal helicase 1, also known as BRCA1-interacting protein 1, BRIP1; GenBank: NM_032043) belongs to a DEAH helicase family and interacts *in vivo *with BRCA1, the protein product of one of the two major genes for hereditary breast cancer susceptibility [1,2]. Interaction is mediated through BRCT domains of BRCA1, motifs that have been shown to be important for the ability of BRCA1 to mediate double-strand break repair and homologous recombination as well as transcription activation [3,4]. The *BACH1 *gene is located at chromosome region 17q23. Besides genes known to be involved in the development and progression of breast cancer, such as *BRCA1 *at 17q21 and *ERBB2 *at 17q12, the presence of other breast cancer associated genes, both tumor suppressors and oncogenes, have been proposed in the long arm of chromosome 17 on the basis of loss of heterozygosity, allelic imbalance, and comparative genomic hybridization studies [5-9]. The possibility of a tumor suppressor gene located distal to *BRCA1 *and involved in both sporadic and hereditary ovarian cancer has also been discussed [10-12].

Previously, Cantor and co-workers have reported on *BACH1 *germ line missense mutations in early-onset breast cancer patients, with one of the patients having a strong family history of both breast and ovarian cancer [2]. In subsequent functional analysis both of the observed mutations, Pro47Ala that is located in a highly conserved nucleotide binding domain and Met299Ile that resides in a helicase homology region, were shown to perturb BACH1 protein function by altering both ATPase and helicase activity [13]. In addition to these rare mutations in individual families, a common Ser-allele of the Ser919Pro polymorphism has recently been associated with an increased breast cancer risk; in a kin-cohort study a 4.5-fold and up to 6.9-fold increased cumulative breast cancer risk was seen for the first degree relatives of Pro/Ser and Ser/Ser carriers vs. Pro/Pro carriers, respectively, by the age of 50 years [14].

Interestingly, biallelic inactivation of *BACH1 *was recently observed in patients with Fanconi Anemia (FA), a recessive chromosomal instability disorder characterized by developmental abnormalities, growth retardation, bone marrow failure, and early predisposition to cancer [15,16]. *BACH1 *mutations were observed in patients with FA complementation group J, whereas similar inactivation of *BRCA2 *has been previously observed in patients with FA complementation group D1 [17]. As individuals with a heterozygous *BRCA2 *mutation are known to have a markedly elevated risk for developing breast cancer, it's tempting to speculate that a similar effect could also be seen with *BACH1*. In epidemiological studies an excess of breast cancer cases, although statistically non-significant, have been observed among FA heterozygotes [18]. However, this observation needs to be taken cautiously due to the small sample size and lack of analyses of individual complementation groups.

In this study, we aimed to evaluate whether there are *BACH1 *genetic variants that contribute to breast cancer risk by screening the *BACH1 *gene for germ line alterations among 43 Finnish *BRCA1/2 *negative breast cancer families. We also evaluated the Ser919Pro variant in a large, independent series of 888 unselected breast cancer patients and in 736 healthy controls.

## Methods

### Breast cancer patients and healthy controls

Breast cancer patients belonging to 43 breast cancer families with at least three breast or ovarian cancer cases in 1^st ^or 2^nd ^degree relatives and with no detectable *BRCA1/2 *mutations were included in the initial mutation analysis. Recruitment of the families through the Department of Oncology, Helsinki University Central Hospital, Finland as well as verification of the cancer diagnoses and exclusion of the *BRCA1 *and *BRCA2 *mutations have been previously described [19-21].

The *BACH1 *variant Ser919Pro was analyzed in a large series of unselected breast cancer patients and healthy controls. The 888 unselected breast cancer patients were collected at the Helsinki University Central Hospital, Finland, during April 1997-March 1998 [22] and January-June 2000 [23], and cover 79% of all consecutive, newly diagnosed breast cancer cases during the collection period. DNA samples from altogether 736 healthy females collected at the same geographical region of Southern Finland were studied as healthy population controls. As the variant was associated with an increased breast cancer risk by the age of 50 years [14] the study cohort as well as the population controls were subgrouped according to the menopausal status (age 50 years was chosen as a surrogate for menopause, and patients with cancer diagnosis at < 50 years were considered premenopausal and ≥50 years as postmenopausal). Breast tumor characteristics (tumor histology, size, and grade; nodal and distal metastasis; estrogen and progesterone receptor status) were available from all patients. Additionally, a Val193Ile variant that was found in only one family in the initial mutation analysis was further genotyped in randomly selected series of 346 familial breast cancer patients and in 183 healthy population controls. All mutation analyses have been performed on DNA samples extracted from peripheral blood.

The study was performed with informed consent from the patients and under appropriate research permissions from the Ethics Committees of the Departments of Obstetrics and Gynecology, and Oncology, Helsinki University Central Hospital, Finland, as well as Ministry of Social Affairs and Health in Finland.

### Mutation analysis

The coding region and exon-intron boundaries of *BACH1 *were screened using conformation sensitive gel electrophoresis (CSGE) with modifications previously described [24]. Samples with aberrant CSGE profiles were reamplified, and the nucleotide changes determined by direct sequencing. Primers and PCR conditions used in the mutation analysis are presented in table 1. The frequency of the Ser919Pro alteration was determined by Amplifluor™ fluorescent genotyping (K-Biosciences, Cambridge, UK). The genotyping for nt c.2755 (codon 919) was successful in 866/888 (97.5%) breast cancer patient samples and in 731/736 (99.3%) healthy control samples. The Val193Ile (c.577G>A) variant was genotyped in 346 (successful in 336, 97.1%) familial breast cancer samples and in 183 (successful in 167, 91.3%) control samples, respectively, by Amplifluor™ fluorescent genotyping as well.

### Statistical analysis

Possible associations between the *BACH1 *Ser919Pro and breast cancer risk, as well as the variant and clinico-pathologic features of the tumors, were tested by univariate analysis. Independent variables were compared with the chi-square test. The mean age at breast cancer diagnoses between the carriers and non-carriers was compared by one-way ANOVA. All p-values were 2-sided, and p-value < 0.01 was considered statistically significant as suggested by Houlston and Peto [25]. All statistical analyses were carried out in the SPSS software (version 12.0 for Windows, SPSS, Chicago, IL, USA).

### Results and discussion

Altogether six germ line *BACH1 *variants were observed in the initial mutation analysis (Table 2). Four of the changes were in the coding region, two of these were missense and two silent substitutions. The missense changes were analyzed further for their possible association with breast cancer risk. The silent alterations have been suggested as neutral polymorphisms by the previous studies [2,26,27], and were not studied here further. The one missense substitution, Val193Ile, was seen in only one family. In addition to the proband diagnosed with breast cancer at the age of 54 years, the variant was also seen in her healthy father and healthy brother, father's sister diagnosed with skin cancer at age 85 years, and the aunts' three children diagnosed with breast cancer at 63 years, ovarian cancer at 59 years, and skin cancer at 64 years, respectively. The grandmother of the proband, whose carrier status is unknown, has been diagnosed with breast cancer at age 74 years. The variant was not observed among 336 familial breast cancer patients or in 167 healthy population controls. It has previously been observed in 3/200 (1.5%) healthy controls and classified as a rare polymorphism [2]. The residue resides in close proximity to the ATP/GTP binding site, and the possible functional significance of this rare variant to BACH1 protein function remains to be determined.

The other observed missense substitution, a common polymorphism Ser919Pro, was associated with an elevated breast cancer risk during the course of this study [14]. In that study the relative cumulative risk by the age 50 years was 4.5 for the female first degree relatives of Pro/Ser heterozygotes (95% CI 0.8–12.2; p = 0.096) and up to 6.9 (95% CI 1.6–29.3; p = 0.018) for the first degree relatives of Ser/Ser homozygotes, when compared to Pro/Pro homozygotes. However, no significant association was seen when the analysis was extended to age 70 years (OR 1.3, 95%CI 0.8–2.8, p = 0.220) [14]. Here we found no association of the variant with breast cancer risk. The odds ratios for the whole study cohort as well as for the pre- and postmenopausal patient groups were close to one both for the Pro/Ser heterozygotes and for the Ser/Ser homozygotes when compared to Pro/Pro homozygotes (Table 3). The alteration did not associate with breast cancer family history (data not shown). No association of the variant with any of the breast tumor characteristics (Table 4) or survival (data not shown) was seen either, and also the age at breast cancer diagnosis was similar in all genotype carrier groups (56.9 years for the Pro/Pro homozygotes, 56.8 years for the heterozygotes, and 56.1 years for the Ser/Ser homozygotes, respectively; p = 0.705). Our data suggest that the *BACH1 *Ser919Pro alteration is not a breast cancer predisposition allele in our study population although a very low risk effect cannot be excluded. A joint effect on breast cancer risk by the Ser919Pro variant and other epidemiological risk factors may also be possible.

Our data, together with previous studies, suggest that germ line mutations in *BACH1 *do not substantially contribute to the remaining proportion of the familial aggregation of breast cancer outside the high-penetrance genes *BRCA1 *and *BRCA2 *[2,26-28]. In the future, it will be interesting to see whether the heterozygous *BACH1 *mutation carriers in FA families have an excess risk of developing breast or other type of cancer. So far, heterozygous mutations of the FA genes, with the exception of *BRCA2*, have been found to be extremely rare, or nonexistent, in breast cancer families [29], and the studied polymorphisms have not been found to confer an increased risk for breast cancer [[30], this study].

## Conclusion

Taken together, our results are in concordance with previous studies where germ line *BACH1 *mutations have been observed in only a very few familial breast cancer patients [2,26-28]. This suggests that even though some functionally deleterious germ line *BACH1 *mutations have been observed in breast cancer patients, such mutations are rare and may account for only a very small proportion, if any, of non-*BRCA1/2 *familial breast cancer. Our results further indicate that *BACH1 *Ser919 is not a breast cancer predisposition allele in the Finnish population.

## Competing interests

The author(s) declare that they have no competing interests.

## Authors' contributions

PV conceived of the study, supervised the molecular genetic studies, performed the statistical analysis and drafted the manuscript. KY and AT carried out the molecular genetic studies. HE and CB collected the patient samples. HN participated in the study design and helped to draft the manuscript.

## Pre-publication history

The pre-publication history for this paper can be accessed here:



## Figures and Tables

**Table 1:** Primers and PCR conditions used in *BACH1 *mutation analysis.

**Exon**	**Strand**	**Primer name**	**Nucleotide sequence (5'→3')**	**Amplicon lenght (bp)**	**Annealing temperature (°C)**
2	Forward	Ex2F	CTGTTTCCAGATTTCTCCC		
	Reverse	Ex2R	GTGAACCCAGAAAATATTCTCC	331	58
3	Forward	Ex3F	CCCTGGAGTGCAATCTCACT		
	Reverse	Ex3RN	TAGCGACAGCATGGCTGAA	319	48
4	Forward	Ex4F	CCTGGGTGAACTGGGCTGTAG		
	Reverse	Ex4RN	TAACAGTAATAATTAAGACTC	316	48
5	Forward	Ex5FN	TTGCCTACCTGTAAGTTATTTATG		
	Reverse	Ex5RN	ACCATGTTCAGCTGTAACTAACTG	233	60
6	Forward	Ex6F	GAGCTGTTTTGGCCTTTGAGA		
	Reverse	Ex6RN	CTGAGTGGGTTGCTACTGTCCT	317	55
7	Forward	Ex7F	GTTCTGATTCCATGTGAGGTT		
	Reverse	Ex7R	GTACATATAAAACACATACTGAGT	448	55
8	Forward	Ex8F	GATGTTCCTCAAATTCTGAGATAA		
	Reverse	Ex8R	CATCTAAAAGCTTTTACATTCAAC	386	55
9	Forward	Ex9F	GCCTATAGTGTGAATTTTAAAATG		
	Reverse	Ex9R	CCTAGTTAACCAAAGTTTACTAAC	395	55
10	Forward	Ex10F	GATCAACGCATGACAATAATGATG		
	Reverse	Ex10R	GGGTTACTCACTAGATTTAATCTG	320	55
11	Forward	Ex11F	GCATGTTTTGTTGGGTTTCATTGT		
	Reverse	Ex11R	GGTATGTATTAAACACATGCTAGC	326	55
12	Forward	Ex12F	GTACCAGCTCTTTCAAATGAG		
	Reverse	Ex12R	CTATCTTTAAAAGAGTCAACCAC	360	55
13	Forward	Ex13FN	GTGCTGGGATTACAGGTGTGAGCCA		
	Reverse	Ex13RN	ACTTGCTGGCACTTCAGGTATCTTC	317	60
14	Forward	Ex14F	CTTGTTGCTTGATCTTTTATGTAC		
	Reverse	Ex14R	CTAGGAAGCTTACTGTGGTAA	356	55
15	Forward	Ex15FN	ACAGCTCTATGAGATATATTG		
	Reverse	Ex15RN	TCATAGGAGAACAAGTACAAT	504	55
16	Forward	Ex16FN	TTACTTAAAGACATTGAACTT		
	Reverse	Ex16RNN	CACTATAAAAGCAAAGCGC	392	54
17	Forward	Ex17FN	CTGTTAGAAGTTAATATGATG		
	Reverse	Ex17RN	GAATACATACCAGTTCCTATG	325	55
18	Forward	Ex18F	CCAATTTTCTGTCTGTCCCAC		
	Reverse	Ex18R	GATAGTAGAGCTCATGTTATGTG	254	55
19	Forward	Ex19F	CTTCACTAGAAAAAGCAAGTG		
	Reverse	Ex19R	CCACCATATTTAAGGAATTAATC	523	48
20A	Forward	Ex20AFN	ACCTAGCAATTATGTTAGCT		
	Reverse	Ex20ARN	TCTGTATCTTCAGGATCGTA	584	48
20B	Forward	Ex20BFN	ATTGATGCCACCCTTACTA		
	Reverse	Ex20BRNN	TAACATAAGCATGATGACATA	565	54

**Table 2:** Observed germ line variants in *BACH1*.

**Exon/Intron**	**Nucleotide change**	**Amino acid change**	**Frequency for heterozygotes (%)**
int5	508-31 C→G	-	11/43 (25.6)
ex6	577 G→A	Val193Ile	1/43 (2.3)
int14	2097+7 G→A	-	1/43 (2.3)
ex19	2637 G→A	Glu879Glu	31/43 (72.1)*
ex19	2755 C→T	Ser919Pro	32/43 (74.4)*
ex20	3411 C→T	Tyr1137Tyr	27/43 (62.8)*

* also homozygotes for the rare allele

**Table 3:** Genotypes of the *BACH1 *Ser919Pro variant among 866 unselected breast cancer patients and 731 healthy population controls.

	**Cases**		**Controls**				
	**n**	**%**	**n**	**%**	OR*	**95% CI**	**p**
**Unselected breast cancer patients, all**	866		731				

Pro/Pro	184	21.2	148	20.2	1		
Pro/Ser	428	49.4	382	52.3	0.901	0.697–1.165	0.427
Ser/Ser	254	29.3	201	27.5	1.016	0.765–1.351	0.911
Pro/Ser + Ser/Ser	682	78.8	583	79.8	0.941	0.738–1.120	0.623
							
**Unselected breast cancer patients, diagnosis <50 years**	276		476				

Pro/Pro	56	20.3	96	20.2	1		
Pro/Ser	140	50.7	249	52.3	0.964	0.653–1.423	0.853
Ser/Ser	80	29	131	27.5	1.047	0.680–1.611	0.835
Pro/Ser + Ser/Ser	220	79.7	380	79.8	0.993	0.686–1.436	0.968
							
**Unselected breast cancer patients, diagnosis ≥50 years**	590		255				

Pro/Pro	128	21.7	52	20.4	1		
Pro/Ser	288	48.8	133	52.2	0.878	0.600–1.289	0.511
Ser/Ser	174	29.5	70	27.5	1.010	0.660–1.545	0.964
Pro/Ser + Ser/Ser	462	78.3	203	79.6	0.925	0.644–1.328	0.671

* as compared to Pro/Pro homozygotes

**Table 4:** Tumor characteristics of the 909 breast tumors from 866 breast cancer patientsanalyzed for the *BACH1 *Ser919Pro variant.

		***BACH1 *Ser919Pro**			
				
	**Total n (%)**	**Pro/Pro**	**Pro/Ser**	**Ser/Ser**	**p**
**Histology**	909				
Carcinoma ductale	713 (78.4)	145 (76.3)	358 (78.7)	210 (79.5)	0.480
Carcinoma lobulare	130 (14.3)	25 (13.2)	70 (15.4)	35 (13.3)	
Carcinoma medullare	13 (1.4)	5 (2.6)	5 (1.1)	3 (1.1)	
Other	53 (5.8)	15 (7.9)	22 (4.8)	16 (6.1)	
					
**Tumor grade**	817				
1	223 (27.3)	51 (29.8)	116 (28.2)	56 (23.9)	0.702
2	353 (43.2)	73 (42.7)	174 (42.2)	106 (45.3)	
3	241 (29.5)	47 (27.5)	122 (29.6)	72 (30.8)	
					
**Tumor size, pT**	887				
1	545 (61.4)	121 (65.8)	269 (60.7)	155 (59.6)	0.423
2	282 (31.8)	47 (25.5)	148 (33.4)	87 (33.5)	
3	31 (3.5)	7 (3.8)	15 (3.4)	9 (3.5)	
4	29 (3.3)	9 (4.9)	11 (2.5)	9 (3.5)	
					
**Lymph node status, pN**	909				
Negative	477 (52.5)	104 (54.7)	223 (49.0)	150 (56.8)	0.101
Positive	432 (47.5)	86 (45.3)	232 (51.0)	114 (43.2)	
					
**Distant Metastasis, pM**	870				
Negative	829 (95.3)	168 (93.9)	416 (96.1)	245 (95.0)	0.478
Positive	41 (4.7)	11 (6.1)	17 (3.9)	13 (5.0)	
					
**Estrogen receptor status**	862				
Positive	680 (78.9)	139 (76.8)	339 (78.5)	202 (81.1)	0.530
Negative	182 (21.1)	42 (23.2)	93 (21.5)	47 (18.9)	
					
**Pogesterone receptor status**	863				
Positive	591 (68.5)	129 (70.9)	289 (66.9)	173 (69.5)	0.577
Negative	272 (31.5)	53 (29.1)	143 (33.1)	76 (30.5)	
